# Mining FDA drug labels using an unsupervised learning technique - topic modeling

**DOI:** 10.1186/1471-2105-12-S10-S11

**Published:** 2011-10-18

**Authors:** Halil Bisgin, Zhichao Liu, Hong Fang, Xiaowei Xu, Weida Tong

**Affiliations:** 1 Department of Information Science, University of Arkansas at Little Rock, 2801 S. University Ave., Little Rock, AR 72204-1099, USA; 2 Center for Bioinformatics, National Center for Toxicological Research, US Food and Drug Administration, 3900 NCTR Road, Jefferson, AR 72079, USA; 3 ICF International at FDA's National Center for Toxicological Research, 3900 NCTR Rd, Jefferson, AR 72079, USA

## Abstract

**Background:**

The Food and Drug Administration (FDA) approved drug labels contain a broad array of information, ranging from adverse drug reactions (ADRs) to drug efficacy, risk-benefit consideration, and more. However, the labeling language used to describe these information is free text often containing ambiguous semantic descriptions, which poses a great challenge in retrieving useful information from the labeling text in a consistent and accurate fashion for comparative analysis across drugs. Consequently, this task has largely relied on the manual reading of the full text by experts, which is time consuming and labor intensive.

**Method:**

In this study, a novel text mining method with unsupervised learning in nature, called topic modeling, was applied to the drug labeling with a goal of discovering “topics” that group drugs with similar safety concerns and/or therapeutic uses together. A total of 794 FDA-approved drug labels were used in this study. First, the three labeling sections (i.e., Boxed Warning, Warnings and Precautions, Adverse Reactions) of each drug label were processed by the Medical Dictionary for Regulatory Activities (MedDRA) to convert the free text of each label to the standard ADR terms. Next, the topic modeling approach with latent Dirichlet allocation (LDA) was applied to generate 100 topics, each associated with a set of drugs grouped together based on the probability analysis. Lastly, the efficacy of the topic modeling was evaluated based on known information about the therapeutic uses and safety data of drugs.

**Results:**

The results demonstrate that drugs grouped by topics are associated with the same safety concerns and/or therapeutic uses with statistical significance (P<0.05). The identified topics have distinct context that can be directly linked to specific adverse events (e.g., liver injury or kidney injury) or therapeutic application (e.g., antiinfectives for systemic use). We were also able to identify potential adverse events that might arise from specific medications via topics.

**Conclusions:**

The successful application of topic modeling on the FDA drug labeling demonstrates its potential utility as a hypothesis generation means to infer hidden relationships of concepts such as, in this study, drug safety and therapeutic use in the study of biomedical documents.

## Background

The number of text documents available from published literature and other public domain repositories is rapidly expanding. Retrieving relevant information from the ever-increasing corpora is a daunting task [1]. One active research area is to extract/discover the relationships of different concepts (e.g., drugs, diseases, and mechanisms) presented in the documents using computational means. For example, Swanson [2] applied Literature Based Discovery (LBD) methods to identify hidden relationships between concepts in the literature. His study demonstrated that although there is no clear relationship between concepts A and C in the literature, their association can be established through concept B that links concepts A and C separately. Swanson studied the relationship between fish-oil (concept A) and Raynaud’s disease (concept C) through blood viscosity (concept B) and suggested that fish-oil could be used for the treatment of Raynaud’s disease.

Another commonly used approach is based on concurrence of terms (e.g., words) in documents, referred to as a “bag of words” or “term frequency and inverse document frequency” (*tf*-*idf*) approach [3]. Gordon and Lindsay [4] replicated the Swanson’s discovery using this approach by mining the Medline database and were able to confirm the fish-oil and Raynaud’s disease relationship. However, such methods like *tf-idf* fail to identify syntactic and semantic relationships between words in the documents. For example, a search for the word “drug” may not return a document containing the word "medicine", although both are used for the same context in most cases. Consequently, *latent semantic indexing* (LSI) was introduced [5], which represents terms and documents as vectors in a concept space by using singular value decomposition (SVD) [3]. Gordon and Dumais [6] employed LSI to uncover the relationship between fish-oil and Raynaud’s disease using the Medline database as a classic case to assess their methodology. The major limitation of LSI is that the derived concepts represented by singular vectors are difficult to interpret.

Recently, topic modeling such as *probabilistic* LSI (*p*LSI) [7] and latent Dirichlet allocation (LDA) [8] have been used widely in the field of computer science with a specific focus on text mining and information retrieval. In topic modeling, documents are a mixture of “topics”, where a topic consists of a set of words that frequently (measured as a probability) occurs together across the documents. The probabilistic nature of topic modeling preserves the contents of the documents, represented by words through topics. In contrast to LBD where the aim is to discover possible association between concepts that represented by the words, topic modeling does not focus on the mutual relations between words. Rather, it uncovers the relationship between topics representing documents via words. Consequently, instead of focusing on co-occurrence and association between words (concepts), topic modeling explores the probabilistic pattern among topics which do not require a transitive relation of words, i.e., A→B→C. Another distinction is that topic modeling is not dependent on the assumption of disjoint literature or datasets while LBD is defined as the process of finding the complementary structures from disjoint science literature.

One major advantage of topic modeling (e.g., *p*LSI) over LSI is that each topic is interpretable in the form of a probability distribution over words. In a study by Blei and Lafferty [9], the authors compared *p*LSI with LDA (a generalization of *p*LSI) by mining articles published in the journal *Science* from 1990-1999. The study suggested that topic modeling can be an effective method to extract meaning from large collections of documents, and that LDA results in more reasonable mixtures of topics in a document compared to *p*LSI. To the best of our knowledge, topic modeling has not yet been extensively investigated in medical and biological sciences [10-14] where textual documents are still the predominant resource used to archive research findings, clinical practices, regulatory actions, etc.

For example, the legally regulated drug labels approved by the Food and Drug Administration (FDA) [15-17] contain valuable information about adverse drug reactions (ADRs) and among other things. The information embedded in these documents are obtained from clinical trials and post-marketing surveillance. The FDA-approved drug labeling text has been a rich resource for study of drug related safety concerns and toxicity, such as drug-induced liver injury [18,19]. For example, studies based on the drug labeling have revealed that drugs receiving black box warnings appear more often in certain therapeutic categories than others [20,21].

**Figure 1:**
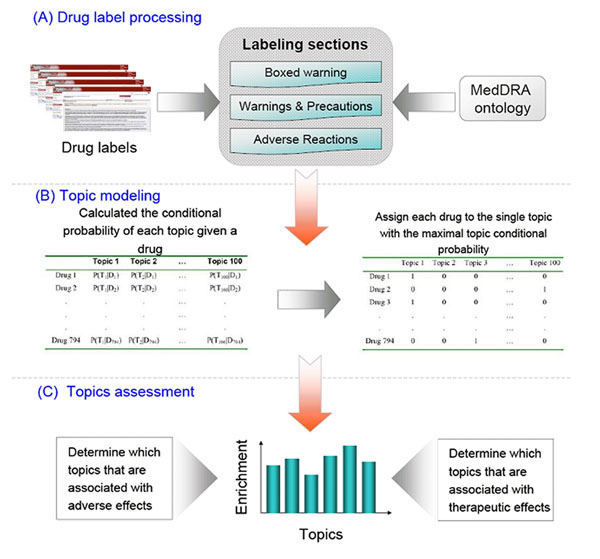
**Overview of the workflow.** The MedDRA ontology was applied to the three drug labeling sections (i.e., Boxed Warnings, Warnings and Precautions, and Adverse Reactions) to generate a list of adverse event terms for each drug, on which topic modeling was applied, followed with statistical analysis to assess the identified topics in the context of safety concern and therapeutic use.

In this study, we evaluated the utility of topic modeling to extract safety information from FDA-approved drug labeling text. Figure 1 shows an overview of our methodology. Our objective is to demonstrate how topic modeling, as an unsupervised learning technique, can contribute as a new venue to the study of drug safety, drug use, and pharmacovigilance. Our results demonstrated that topic modeling can group and classify drugs based on their shared commonalities (such as safety profiles and therapeutic uses) with no need of *a priori* knowledge, and thus holds the potential for broad applications in biomedical research, particularly for the FDA documents.

## Methods

### Drug label data set

The FDA drug labeling text was obtained from DailyMed (http://dailymed.nlm.nih.gov/dailymed/), where most FDA-approved prescription drug labels were available. We noticed that a drug was often associated with multiple labels, different names could be used for the same drug, or the administration route could vary for the same drug. Thus, a set of inclusion/exclusion criteria were used for the preprocessing of the drug labels to generate a “one drug with one label” data set: 1) the labels for the same drug were grouped together using the generic name; 2) for each drug, the latest version of the drug label was used according to its effective date; 3) drugs containing more than one active ingredient were excluded; 4) only small molecular drugs were included; and 5) only prescription drugs with tablet or capsule types and intravenous routes were considered.

After the preprocessing, 794 unique drugs remained. Thirty-five percent (279) of these had a Boxed Warning (BW), the most severe label associated with ADRs. The BW information was used to assess the performance of topic modeling for generating topics related to safety concerns. In addition, 635 drugs out of 794 drugs can be defined by the first level of the Anatomical Therapeutic Chemical Classification System (ATC) (http://www.who.int/classifications/atcddd/en/). These data were used to evaluate topic modeling’s performance in the identification of topics related to therapeutic use.

### Data processing

The extracted XML formatted labels of 794 drugs were parsed. Three labeling sections, namely BW, Warnings and Precautions (WP), and Adverse Reactions (AR) were used for further analysis. These three sections contain the labeling information about safety concerns, adverse events, and cautions that should be considered in the clinical use of the drug. We filtered the raw text based on the standard ADR terms of the Medical Dictionary for Regulatory Activities (MedDRA) (http://www.meddramsso.com/), and created separate text documents for every drug. Specifically, the lowest level of terms in the MedDRA database was used with 68,259 terms from 26 organs [22]. Consequently, we built an ADR profile for each drug with the standard terms from MedDRA, which was the input for the following topic modeling.

### Topic modeling

Topic modeling is based on the idea that a document is a mixture of topics, and that each word is selected with a probability given one of the document topics. More specifically, let *P*(*z*) be the distribution of topics for a given document, and *P*(*w|z*) be the probability distribution over words *w* given topic *z*. Then each word *w_i_* (where *i* is the index for *i*-th word) of a document is generated in two steps: first, a topic *j* is selected with a probability of *P*(*z_i_*=*j*) following the probability distribution *P*(*z*); second, a word *w_i_* is picked out with a probability of *P*(*w_i_* | *z_i_*=*j*). Then the two-step generative process specifies the following distribution of words in a document:

where *T* represents the number of topics.

For document *d*, *θ*^(^*^d^*^)^ = *P*(*z*) stands for the multinomial distribution over topics. In the *p*LSI model, there are no assumptions on how the *θ*’s are generated [7]. The LDA model by Blei and Lafferty [9] is a generative model, where a Dirichlet prior on *θ* makes not only the inference step more convenient, but also the model more generative for new documents [8].

In this study, we used the LDA approach to obtain the parameter *θ* for every document (i.e., the drug labels). The topics were extracted by using Mallet, an open source software package from UMASS [23]. We used 100 as the number of topics to carry out the analysis and calculated the conditional probability of each topic given a drug, as illustrated in Figure 1 (the left table in the middle panel). Since it is a probability distribution over topics, the row-wise summation is equal to 1.

### Drug grouping

The topic distribution measures the connection (or relatedness) of a drug with a specific topic (i.e., the conditional probability of topic for a given drug as shown in the table on the left of Figure 1). We used this statistical probability to group the drugs by associating them with topics. More specifically, the drugs were assigned to the most probable topics for the given drugs. The result of this topic assignment is illustrated in the table on the right of Figure 1, where each row has exactly one entry with the value 1, which indicates the assigned topic for the given drug (all other entries are 0). In the case of a tie, we arbitrarily assigned the drug to any of the topics with maximal conditional probability of topics for given drugs.

## Results

Figure 1 illustrates the flowchart of this study. The 794 FDA-approved drug labels were processed and the three labeling sections (i.e., BW [24], WP[17], and AR [15,25]) were extracted. Then, the MedDRA ontology was used to convert the free text of each label to standard ADR terms. Afterwards, topics were generated by using LDA followed by assigning each drug to the most probable topic. Finally, the efficacy of the topic modeling was evaluated based on the ATC and BW labels of the drugs to examine the relationship of the identified topics with therapeutic use and safety, respectively.

**Figure 2:**
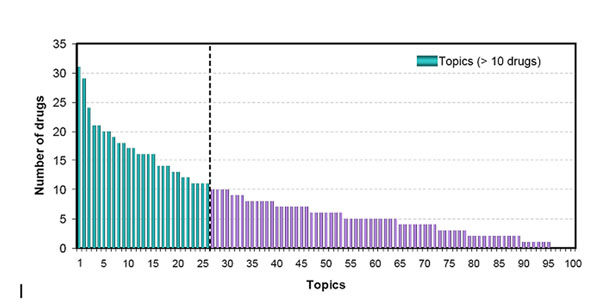
**The distribution of the number of drugs in the 100 topics.** The cutoff for topics to perform further analysis on was set at 10 drugs and is shown on the graph.

The analysis resulted in 100 topics that were ranked according to the number of drugs in each topic, as depicted in Figure 2. We analyzed the common properties shared by the drugs in each topic. In order to achieve a meaningful statistical test and to avoid bias, we chose topics containing at least 10 drugs for assessing their relevance to therapeutic uses and safety concerns by using ATC and BW labels, respectively. A total of 27 topics (the first 27 bars in Figure 2 and Additional file 1) were selected for the following enrichment analysis.

### Topic analysis in terms of BW

BW has been defined by the regulatory document (21CFR201.57) as “certain contraindications or serious warnings, particularly those that may lead to death or serious injury.” It is the strongest medication-related safety warning that the FDA can issue for a prescription drug, and such a decision issued by the FDA has serious implications for the licensed practitioner, the pharmacist, the patient, the pharmaceutical manufacturer, and the distributor [18,19]. Thus, a topic populated with BW drugs is likely related to drug safety.

**Figure 3:**
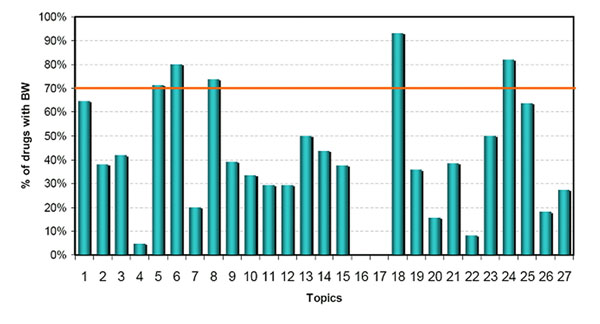
**The percentage of drugs with Boxed Warning (BW) for 27 topics.** This percentage was calculated for each of 27 topics that contain at least 10 drugs.

There were 455 drugs involved in the aforementioned 27 topics (57% of the total 794 drugs in 100 topics). Among these 455 drugs were 188 that had BW (41%). Figure 3 shows the percentage of BW drugs in each of the 27 topics. Five topics (5, 6, 8, 18, and 24) contained at least 70% of the drugs with BW in each topic. Table 1 shows the statistical analysis of these five topics by using Fisher’s exact *t* test. All five topics had a *p*-value of less than 0.05. The content varied between topics; for example, drugs grouped in topic 24 are related to liver injury, while those classified under topic 5 could cause kidney injury.

**Table 1:** Five topics with highly populated BW drugs (>70%)

Topics	Statistics				Corresponding ADRs from MedDRA
		
	# drugs	# BW Drugs	%	P-value	
Topic 5	21	15	71%	0.0121	Creatinine; renal-failure; hyperkalemia; potassium; injury
Topic 6	20	16	80%	0.0014	Suicide; irritability; restlessness; agitation; anxiety
Topic 8	19	14	74%	0.01	Bleeding; stroke; gastrointestinal-bleeding; myocardial-infarction; coronary artery bypass
Topic 18	14	13	93%	2.38E-4	Death; psychosis; elderly; dementia; extrapyramidal symptoms
Topic 24	11	9	82%	0.0135	Hepatitis; hepatotoxicity; hepatic-failure; injury; death

The results indicated that topic modeling can yield statistically significant topics that group and identify drugs with severe safety concerns using FDA-approved drug labels. Next, we further assessed the performance of the topic modeling in deriving topics that represent therapeutic uses of drugs.

### Topic analysis in terms of therapeutic use

**Figure 4:**
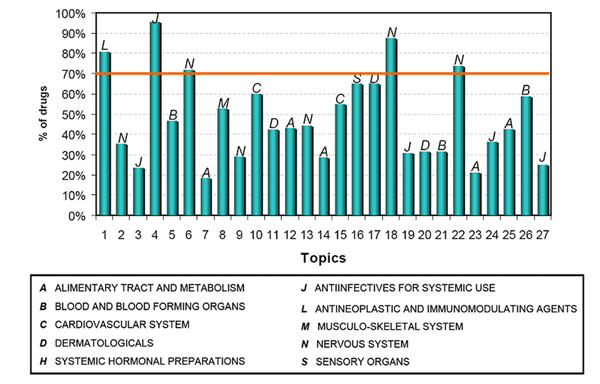
**The purity of the top therapeutic category for 27 topics.** Each of 27 topics was assigned to one therapeutic category according to which ATC category contained the most drugs from that topic; the percent of drugs belonging to that category from the topic is shown.

The aforementioned analysis procedure was similarly conducted to investigate whether topic modeling can group and classify different drugs according to their therapeutic use. The drugs were mapped to the first level of ATC codes. The 455 drugs in the 27 topics covered all 14 categories of the first level of ATC. Five topics (1, 4, 6, 18, and 22) were identified as containing >70% drugs belonging to at least one of the 14 ATC categories (Figure 4) with statistical significance (*p* < 0.05, Table 2). These topics were related to drugs used for Nervous System disorders (topics 6, 18, and 22), Antiinfectives for Systemic Use (topic 4), and Antineoplastic and Immunomodulating Agents (topic 1).

**Table 2:** Five topics with highly populated drugs (>70%) in a therapeutic category

Topics	Statistics				Corresponding ADRs from MedDRA
		
	# drugs	Therapeutic categories (First Level of ATC)	%	P-value	
Topic 1	31	Antineoplastic and immunomodulating agents (L/24)	81%	0.0005	Neutropenia; stomatitis; infection; myelosuppression; sepsis; immunosuppression
Topic 4	21	Antiinfectives for systemic use (J/19)	96%	0.0005	Colitis; diarrhea; allergy; colectomy; eosinophilia
Topic 6	20	Nervous system (N/14)	73%	0.0011	Suicide; irritability; restlessness; agitation; anxiety
Topic 18	14	Nervous system (N/11)	88%	8.31E-004	Death; psychosis; elderly; dementia; extrapyramidal symptoms
Topic 22	12	Nervous system (N/8)	75%	0.020	Depression; excitement; dysphoria; unconsciousness; sleep disturbances

We listed the ADR terms for each of five topics in Table 2 to examine which side effects might arise from medications in these topics. We found that there exists a distinct difference in adverse reactions for topics 6, 18 and 22 although they are all used for Nervous System disorders. The drugs in topic 18 had a significant impact on elderly persons with dementia and may cause death, while the drugs in topic 6 could cause anxiety, irritation, and even suicide attempts. Compared to topics 6 and 18, the side effects for the drugs in topic 22 were much milder, and include sleep disturbance and depression.

## Discussion

Techniques such as bag of words and latent semantic analysis have been commonly used in text mining. Recently, topic modeling has proven to be a robust approach for text mining with distinct advantages over other approaches. In this study, we made a first attempt to apply topic modeling to FDA-approved drug labels. Our approach consisted of two main steps. First, for each drug, we inferred the topics from a mixture of standard adverse reaction terms. Second, the drugs were grouped based on the shared topics. Our results demonstrate that topic modeling offers several distinct advantages, particularly when applied to drug labels. First, as an unsupervised learning technique, it does not require any training data or *a priori* knowledge about drug labels. Second, it can discover the gist and hidden patterns in the labels. Furthermore, the discovered topics can be successfully used for grouping and identifying drugs within the same therapeutic category as well as drugs with severe safety concerns. Last but not least, the discovered topics can be easily interpreted by using some domain specific knowledge.

According to the theory of topic modeling, each topic represents certain common properties, which reflects the pattern in the free texts. The topics populated with more drugs do not necessarily correlate with the degree of shared commonality among drugs. Finding out the exact meanings of the topics requires additional information and domain knowledge. In this study, we evaluated whether the topic modeling can generate biologically relevant topics from the drug labeling text. Since the drug labeling text contains information largely related to safety and therapeutic use, we tested the topic modeling using the drugs with ATC and BW labels.

We identified five topics each that were significant in the therapeutic application and safety concerns of drugs, respectively. Most topics of safety concern are different from these for therapeutic application except two; topics 6 and 18 were significant in both therapeutic use and safety. In other words, the drugs in both of these topics are not only used for the same therapeutic purpose, but the adverse reactions related to this medication are so severe that their uses need to be regulated with BW. This result also indicates that a topic is not necessary associated with only one concept, and it could be related to several commonalities shared by the drugs. However, in order to discern the hidden meanings of a topic, careful analysis and domain specific knowledge are required, indicating that topic modeling can be a powerful hypothesis generation tool to guide systemic investigation of the relationship between the topics and downstream biological actions.

We also observed that different topics might fall into the same therapeutic category but with different biological indication. Specifically, topics 6, 18, and 22 all belong to the same therapeutic category, i.e., Nervous System disorders. However, the degree of severity in adverse reaction and the target population for application are distinctly different between drugs in different topics. The drugs in topics 6 and 18 could associate with more serious adverse events than those in topic 22. For instance, Mirtazapine, a drug in topic 6, is an antidepressant drug with BW [26]. Compared to placebo, it increases the risk of suicidal thinking and behavior (suicidality) in children, adolescents, and young adults in short-term studies of major depressive disorder and other psychiatric disorders. This is consistent with our findings using the unsupervised topic modeling method.

Understanding drug safety and efficacy continue to be critical and challenging issues for academia, government agencies, and pharmaceutical companies in their mutual goal to improve patient health. FDA drug labels contain comprehensive information for prescription drugs about their safety and therapeutic use, which is a rich resource to guide the development of new methodologies to understand underlying mechanisms of drug toxicity and efficacy. Given the fact that the labeling is constantly changing in light of new data available for a drug and that the number of labels will continually increase when new drugs are brought into the market, we need to have an accurate and effective methodology to mine this rich and constantly evolving resource. Our first attempt of applying topic modeling to the information contained in FDA drug labels reveals its ability to group drugs together based on similar intrinsic properties such as the patterns in therapeutic use and safety, which could be used to study modes of action of the grouped drugs. We believe that topic modeling also holds potential for mining other biological documents, e.g., the FDA’s Adverse Event Reporting System (AERS), PubMed literature, documents from the tobacco industry, Online Mendelian Inheritance in Man (OMIM) [27,28], and GeneRIF [29].

## Conclusions

This study investigates the efficacy of topic modeling for the discovery of hidden patterns and their meanings from FDA-approved drug labels. The results demonstrate that drug groups based on topics are statistically significantly enriched in terms of either drug safety categories or therapeutic categories. Topic modeling could thus offer a novel way to discern inter-relationships among drug, target, ADR, gene, pathway, and disease data from public biomedical literature and drug databases. We conclude that topic modeling is a promising unsupervised learning technique for mining biomedical documents by retrieving, organizing, and integrating information from a textual database for drug safety, pharmacovigilance, and drug repositioning.

## Disclaimer

The views presented in this article do not necessarily reflect those of the US Food and Drug Administration.

## List of abbreviations used

FDA: Food and Drug Administration; ADR: adverse drug reaction; MedDRA: Medical Dictionary for Regulatory Activities; LDA: latent Dirichlet allocation; LBD: Literature Based Discovery; LSI: latent semantic indexing; pLSI: probababilistic latent semantic indexing; SVD: singular value decomposition; BW: Boxed Warning; WP: Warnings and Precautions; AR: Adverse Reactions; ATC: Anatomical Therapeutic Chemical Classification System; AERS: Adverse Event Reporting System; OMIM: Online Mendelian Inheritance in Man.

## Competing interests

The authors declare that they have no competing interests.

## Authors' contributions

HB and ZL, performed all calculations and data analysis, and wrote the first draft of manuscript. WT and XX developed the methods and had the original idea and guided the data analysis and presentation of results. HF contributed to the data analysis, verified the calculations, and assisted with writing the manuscript. All authors read and approved the final manuscript.

## Supplementary Material

Additional file 1 Drug list for 27 topics. Click here for file
