# Supplementary material for: Mining FDA drug labels using an unsupervised learning technique - topic modeling
Source: BMC Bioinformatics. 2011 Oct 18;12(Suppl 10):S11. doi: 10.1186/1471-2105-12-S10-S11 (PMC3236833; doi:10.1186/1471-2105-12-S10-S11)
Supplement: Additional file 1 — Drug list for 27 topics. [file 1471-2105-12-S10-S11-S1.doc]

Table S1 Drug list for 27 topics

| ***Topics*** | ***No. of drugs*** | ***Drug list*** |
| --- | --- | --- |
| ***1*** | 31 | Alemtuzumab; Azathioprine; Cladribine; Cyclosporine; Cytarabine; Dactinomycin; Daunorubicin; Dexrazoxane; Dolasetron;  Doxorubicin; Epirubicin; Erlotinib; Etoposide; Filgrastim; Fludarabine; Fluorouracil; Gefitinib; gemtuzumab ozogamicin; Idarubicin; Ifosfamide; Imatinib; Irinotecan; Marinol; Mebendazole; Mycophenolate mofetil; Pentostatin; pralatrexate; Tacrolimus; Thioguanine; Tositumomab; Vinorelbine |
| ***2*** | 29 | Alfentanil; Atracurium; beractant; dalteparin sodium; Desflurane; Dinoprostone; Doxacurium; Doxacurium chloride; echothiophate iodide ; Enflurane; Enoxaparin; Etomidate; fospropofol disodium; Galantamine; Hexachlorophene; Isoflurane; maraviroc; Meropenem; metyrosine; Midazolam; Oxymetazoline; Pancuronium; Remifentanil; Rocuronium; Sevoflurane; Succinylcholine; tinzaparin sodium; Vecuronium; Vigabatrin |
| ***3*** | 24 | Allopurinol; Aminosalicylic Acid; Carbamazepine; Cephalexin; Chlorambucil; Chloroquine; Chlorpropamide; Cysteamine; Dapsone; Diclofenac; etravirine; Famotidine; Flucytosine; Fluvastatin; glyburide; Indapamide; Penicillamine; Phenytoin; Piperacillin; Propylthiouracil; Ramipril; Rifampin; Sulfisoxazole; Valdecoxib |
| ***4*** | 21 | Amoxicillin; Ampicillin; Aztreonam; Carbenicillin; Cefadroxil; Cefazolin; Cefoperazone; Cefotaxime; Cefotetan; Cefoxitin; Ceftibuten; Ceftizoxime; Ceftriaxone; Cefuroxime; Cephradine; Clarithromycin; indocyanine green; lincomycin; Loracarbef; Nitrofurantoin; Trimethoprim |
| ***5*** | 21 | Benazepril; Caffeine; Candesartan; Captopril; Darbepoetin alfa; Eplerenone; Epoetin alfa; Eprosartan; Exenatide; Fosinopril; Guanidine; Immune globulin; Irbesartan; Losartan; Mitomycin; Ondansetron; Perindopril; Spironolactone; Telmisartan; Triamterene; Vancomycin |
| ***6*** | 20 | Amantadine; Amitriptyline; Amoxapine; betaine; Citalopram; Clomipramine; Desipramine; Doxepin; Fluoxetine; Fluvoxamine; Imipramine; Interferon Alfa-2a; Isocarboxazid; Mirtazapine; Phenelzine; Pseudoephedrine; ramelteon; Tranylcypromine; Trimipramine; Varenicline |
| ***7*** | 20 | Anakinra; Anastrozole; Bisoprolol; Diltiazem; Epinastine; Escitalopram; Exemestane; Fosfomycin; Imiquimod; levalbuterol hydrochloride; Lovastatin; Nelfinavir; omega-3-acid ethyl esters; Pemirolast Potassium; Pentosan Polysulfate; Quinapril; Rasagiline; Repaglinide; ursodiol; zileuton |
| ***8*** | 19 | Celecoxib; Etodolac; Fenoprofen; Flurbiprofen; Ibuprofen; Indomethacin; Ketoprofen; Ketorolac; Meclofenamate Sodium ; Mefenamic acid; Meloxicam; Naproxen; Nateglinide; Oxaprozin; prasugrel hydrochloride; Progesterone; Reteplase; Tolmetin; Tranexamic Acid |
| ***9*** | 18 | Baclofen; Benztropine; Biperiden; Ciprofloxacin; Cycloserine; Diazepam; Diphenoxylate; Flurazepam; Lorazepam; Meperidine; Mexiletine; Nabilone; Oxazepam; Procarbazine; Promethazine; Selegiline; Tizanidine; Triazolam |
| ***10*** | 18 | Acebutolol; Amlodipine; Atenolol; Betaxolol; Doxazosin; enalaprilat; Epinephrine; Isosorbide Dinitrate; Methylergonovine maleate; Metoprolol; Nadolol; nebivolol hydrochloride; Nitroglycerin; Phentolamine; regadenoson; Timolol; Verapamil; Vinblastine |
| ***11*** | 17 | Alitretinoin; Bosentan; Chlordiazepoxide; Choriogonadotropin alfa; chorionic gonadotropin ; Dextromethorphan; Enalapril; follitropin beta; ganirelix acetate; Interferon alfacon-1; Isotretinoin; lubiprostone; Lutropin alfa; olmesartan medoxomil; Thalidomide; thiotepa; Urofollitropin |
| ***12*** | 17 | Bumetanide; Capreomycin; Ceftazidime; Chlorzoxazone; Clotrimazole; Colestipol; deferasirox; Docetaxel; edetate calcium disodium; eltrombopag olamine; Famciclovir; Gemcitabine; Mecasermin; mesalamine; Pravastatin; Trimetrexate; zinc acetate |
| ***13*** | 16 | Amifostine; Buprenorphine; edrophonium chloride ; Fentanyl; halothane; Hydromorphone; Lidocaine; Methadone; Methohexital; nalmefene hydrochloride; Naloxone; Norepinephrine; Oxycodone; Propofol; Propoxyphene; tapentadol hydrochloride |
| ***14*** | 16 | Abatacept; Agalsidase beta; Clofarabine; denosumab; eculizumab; Ezetimibe; Fexofenadine; Formoterol; Ibritumomab; Laronidase;  Natalizumab; Oxybutynin; Rosiglitazone; saxagliptin; Valsartan; velaglucerase alfa |
| ***15*** | 16 | Adapalene; Apraclonidine; Bimatoprost; Brimonidine; Brinzolamide; bromfenac sodium; Cyclopentolate; Dorzolamide; Ketotifen; Latanoprost; Loteprednol Etabonate; Malathion; Medrysone; Nedocromil; sulconazole nitrate; sulfaccetamide sodium |
| ***16*** | 16 | Adenosine; Digoxin Immune Fab; Dihydroergotamine; Dobutamine; Dofetilide; Eletriptan; Flecainide; Frovatriptan; Ibutilide; Milrinone; moricizine hydrochloride; Naratriptan; nilotinib; Sotalol; Sumatriptan; Zolmitriptan |
| ***17*** | 14 | Alclometasone; Amcinonide; Clobetasol; Clocortolone; Desoximetasone; Fluocinolone Acetonide; Fluocinonide; halcinonide; Halobetasol Propionate; hycrocortisone; Mometasone; Naftifine; Oxiconazole; Prednicarbate |
| ***18*** | 14 | Chlorpromazine; Clozapine; Haloperidol; Lisinopril; Loxapine; Metoclopramide; Paliperidone; Prochlorperazine; Quetiapine; Rubidium Rb-82; Thioridazine; thiothixene; Trifluoperazine; Ziprasidone |
| ***19*** | 14 | Carvedilol; Darunavir; Diazoxide; Fosamprenavir; Glimepiride; Glipizide; lanreotide acetate; Metformin; Nelarabine; pramlintide acetate; Probenecid; sorbitol; Tolazamide; Tolbutamide |
| ***20*** | 13 | Aminocaproic Acid; Coagulation Factor IX human; erythropoietin; estropipate; fibrinogen; floxuridine; Heparin; Idursulfase; Mannitol; Mephenytoin; Raloxifene; Saquinavir; Toremifene |
| ***21*** | 13 | Aminoglutethimide; Betamethasone; cortisone acetate; Danazol; Fludrocortisone; Hydrocortisone; levothyroxine sodium ; Lithium; methylprednisole; Methylprednisolone; Naphazoline hydrochloride; Nicardipine; Prednisone |
| ***22*** | 12 | Alprazolam; Chloroprocaine; dexmethylphenidate hydrochloride; Estazolam; Eszopiclone; Mepivacaine; methamphetamine hydrochloride; Methylphenidate; Pentobarbital; Prilocaine; Ropivacaine; Secobarbital |
| ***23*** | 12 | Abacavir; Acetazolamide; Adefovir Dipivoxil; Amiloride; Emtricitabine; Glycine; Lamivudine; Methazolamide; Potassium Chloride; Stavudine; tenofovir disoproxil ; Zidovudine |
| ***24*** | 11 | Asparaginase; chenodiol; Efavirenz; Ketoconazole; Labetalol; Naltrexone; Nefazodone; Nevirapine; Pemoline; Telithromycin; Terbinafine |
| ***25*** | 11 | Amikacin; Carboplatin; Cisplatin; colistimethate; Gentamicin; Kanamycin; Magnesium Sulfate; Neomycin; Tobramycin; Vardenafil; zoledronic acid |
| ***26*** | 11 | Bethanechol; Buspirone; capsaicin; Esmolol; Fenoldopam; glucagon hydrochloride; Hydroxocobalamin; Ketamine; Lepirudin; Levobunolol; Treprostinil |
| ***27*** | 11 | alvimopan; calcipotriene; Fluconazole; Modafinil; Orlistat; Oseltamivir; Panitumumab; Pralidoxime; Ranolazine; sodium phenylbutyrate; Vincristine |
